# Contribution of ^18^F-FDG PET/CT to Staging of Head and Neck Malignancies

**DOI:** 10.4274/mirt.51423

**Published:** 2018-02-01

**Authors:** Yeşim Ceylan, Özgür Ömür, Filiz Hatipoğlu

**Affiliations:** 1 Adıyaman Faculty of Medicine, Training and Research Hospital, Clinic of Nuclear Medicine, Adıyaman, Turkey; 2 Ege University Faculty of Medicine, Department of Nuclear Medicine, İzmir, Turkey; 3 Bilim Spect Nuclear Medicine Center, İzmir, Turkey

**Keywords:** Head and neck cancer, PET/CT, ^18^F-FDG

## Abstract

**Objective:**

Accurate staging of head and neck cancer (HNC) plays an important role in patient management as well as protection of functional characteristics of the head and neck region. Our aim was to investigate the contribution of 2-[^18^F]-fluoro-2-deoxy-d-glucose (FDG) positron emission tomography/computed tomography (PET/CT) as part of HNC staging to clinical evaluation and treatment planning.

**Methods:**

Clinical records of 138 HNC cases who has undergone ^18^F-FDG PET/CT imaging were retrospectively reviewed. Sixty-five cases who had accessible clinical follow-up data were included in the study group, and their PET/CT and conventional imaging findings were evaluated.

**Results:**

In the case group with a PET/CT and magnetic resonance imaging (MRI) for evaluation of primary lesion the sensitivity rates for PET/CT and MRI were calculated as 91.3% and 82.6%, the positive predictive values (PPV) as 91.3% and 82.6%, specificity as 71.4% and 42.8%, and the negative predictive value (NPV) as 71.4% and 42.8%, respectively. In terms of metastatic lymph node evaluation, the sensitivity was calculated as 100% and 88.8%, the NPV as 100% and 83.3%, respectively. The PPV and specificity was 100% for both modalities. In the case group with CT for primary lesion evaluation, the sensitivity and PPV were found as 95.2% and 100% for PET/CT, and as 85.7% and 94.7% for CT, respectively. in metastatic lymph node evaluation, the sensitivity was found as 100% for PET/CT and 50% for CT, and the PPV, specificity and NPV were determined as 100% for both methods. PET/CT findings resulted in a change in ‘tumor, node, metastasis’ staging in 5 cases.

**Conclusion:**

PET/CT in HNC contributes to staging, thus playing a role in treatment planning, especially in patients with locally advanced disease.

## Abstract (tr)

**Amaç:**

Baş ve boyun kanserlerinde (BBK) doğru evreleme, uygun tedavi modalitesinin seçilmesi ve baş boyun yapılarının işlevsel özelliklerinin korunmasında önemli rol oynamaktadır. Çalışmamızda BBK’lerin evrelendirilmesinde 2-[^18^F] floro-2-deoksi-d-glukoz (^18^F-FDG) pozitron emisyon tomografisi/bilgisayarlı tomografi (PET/BT) görüntülemenin klinik değerlendirme ve tedavi planlamasına katkısı araştırıldı.

**Yöntem:**

^18^F-FDG PET/BT görüntüleme uygulanan 138 BBK’lı olgunun klinik kayıtları retrospektif olarak incelendi. Klinik izlem verilerine ulaşılabilen 65 olgu çalışma grubuna alındı ve olguların PET/BT ve konvansiyonel görüntüleme bulguları değerlendirildi.

**Bulgular:**

Primer lezyon değerlendirmede PET/BT ve manyetik rezonans görüntülemesi (MRG) olan olgu grubunda PET/BT ve MRG için sırasıyla sensitivite %91,3, %82,6, pozitif prediktif değer (PPD) %91,3 ve %82,6, spesifite %71,4 ve %42,8 ve negatif prediktif değer (NPD) %71,4 ve %42,8 olarak hesaplandı. Metastatik lenf nodu değerlendirmede ise PET/BT ve MRG için sırasıyla sensitivite %100 ve %88,8, NPD %100 ve %83,3, PPD ve spesifite ise her iki yöntem için de %100 olarak hesaplandı. BT’si olan olgu grubunda primer lezyon değerlendirmede sensitivite PET/BT için %95,2, BT için %85,7, PPD %100 ve %94,7 olarak bulundu. Metastatik lenf nodu değerlendirmede sensitivite PET/BT için %100, BT için %50, PPD, spesifite ve NPD %100 olarak bulundu. PET/BT bulguları 5 olguda ‘tümör, nod ve metastaz’ evrelemesinde değişikliğe neden oldu.

**Sonuç:**

PET/BT BBK’da evrelemeye katkıda bulunarak özellikle lokal ileri evredeki olgularda tedavi planlamasında önemli rol oynar.

## INTRODUCTION

Thorough evaluation of patients with head and neck cancer (HNC), which include malignancies originating from the paranasal sinus, nasal-oral cavity, pharynx and larynx, is fundamental in order to apply the most appropriate treatment while optimizing functional and cosmetic outcomes. Ultrasound, computed tomography (CT) and magnetic resonance imaging (MRI) are used prior to selecting treatment modality as well as endoscopic evaluation. CT is especially useful in the evaluation of lung metastasis and local or metastatic bone involvement. However, it is generally insufficient in determining cartilage invasions and in differentiating tissue thickening from tissue damage caused by the tumor (1). MRI is superior to CT in terms of local evaluation of the primary tumor by showing bone marrow involvement (2). Nevertheless, conventional imaging methods are inadequate in small or localized submucosal tumors in determining relapse or residual lesion after radiotherapy (RT) and surgery (3). In addition, the presence of morphologic criteria suggesting lymph node metastasis such as central necrosis, early contrast enhancement, irregular boundary and circular shapes, do not have sufficient specificity in diagnosing metastases. Thus, 2-[^18^F]-fluoro-2-deoxy-d-glucose (^18^F-FDG) positron emission tomography (PET)/CT, which provides both metabolic and anatomical information and simultaneously evaluates local tumor and distant metastases, is required for accurate staging (4).

In this study, it was aimed to investigate the contribution of ^18^F-FDG PET/CT imaging method as part of HNC staging to clinical evaluation and treatment planning.

## MATERIALS AND METHODS

### Patient Group

**Figure 1:**
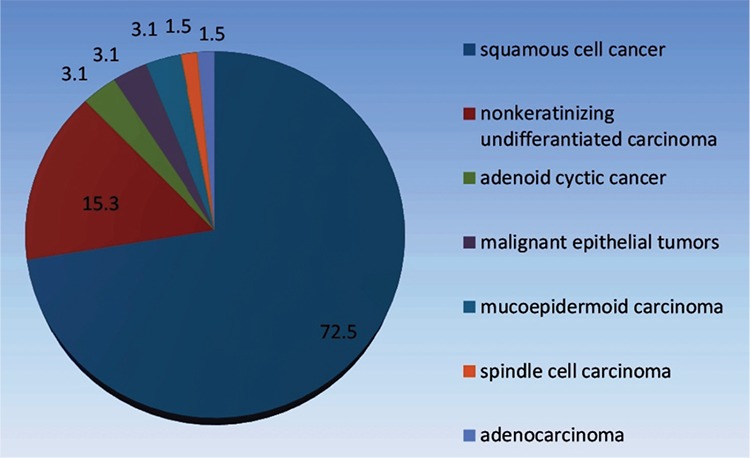
Percentage distribution of the case group according to tumor histopathology

The study protocol was approved by the Ege University Faculty of Medicine, Ethical Committee of Clinical and Laboratory Research Department (Date 24 January 2014, Protocol number: 14-1.1/2). The study was performed in accordance with the ethical standards laid down by Declaration of Helsinki in 1964 and all its subsequent revisions. The clinical records of 138 HNC cases who were referred to the Department of Nuclear Medicine between 2 January 2012 and 1 November 2013 were examined. Our study group consisted of 65 patients who has undergone whole-body PET/CT imaging and whose follow-up data were accessible. ^18^F-FDG PET/CT imaging was performed for staging in 37 cases and re-staging in 28 cases. Forty-nine (75.3%) patients were male and 16 (24.7%) were female. Their mean age was 68±7.07 years (age interval: 18–89 years). Percentage distribution of the patients in the case group according to primary tumor histopathology is shown in Figure 1.

### Patient Preparation and PET/CT Imaging

Before the PET/CT study, detailed clinical information of the patients and informed consent were obtained. All patients fasted for 6 hours. If the blood glucose level was under 200 mg/dL, 7-15 mCi (259-555 MBq) ^18^F-FDG was administered intravenously. Patients then rested in a quite dark room just prior to the exam. Approximately 45-90 minutes after the injection, image acquisition was performed.

^18^F-FDG PET/CT imaging was performed by using the Siemens Biograph 16 TruePoint PET/CT scanner (Siemens Medical Solutions, Inc. USA) with a slice thickness of 5 mm. PET/CT data were acquired from the vertex to the proximal thighs in supine position with the arms raised over head, and additional head and neck images were acquired with the arms down. For attenuation correction and anatomic correlation, CT data was obtained from the vertex to the upper thighs (130 keV, 120 mA) with a rotation time of 0,6 sec and a slice thickness of 5 mm, 1 mm/sec bed speed. Immediately after CT data acquisition, PET scanning was performed in 2D mode with a scan duration of 1.8 min per bed. PET data were reconstructed iteratively (matrix size 512x512) with ordered subsets expectation maximization algorithm (3 iteration, 21 subset).

PET/CT data were examined on Syngo MM Workstation. Images were evaluated qualitatively and quantitatively. Two nuclear medicine specialists examined the PET/CT images for qualitative evaluation. According to visual analysis, the reports were defined as ‘abnormal’ when ^18^F-FDG-uptake was greater than background blood pool activities or surrounding normal tissue. For quantitative analysis of PET images, region of interest was drawn around the most intense ^18^F-FDG uptake and maximum standard uptake value (SUV_max_ corrected for body weight) was calculated.

### Data Analysis and Statistics

SPSS package program (Statistical Package for the Social Sciences) version 18.0 (SPSS Inc., Chicago, Illionis, USA) was used for statistical analysis. Mann-Whitney U test was used for evaluation of differences in SUV_max_ between malignant and benign lesions, and McNemar test was used in comparison of the results of conventional methods. P value of <0.05 was considered as significant.

## RESULTS

The average SUV_max_ of primary lesions and metastatic lymph nodes in study patients are shown in Table 1. The difference between the SUV_max_ of malignant and benign lesions that were confirmed histopathologically in the primary tumor sites (p=0.013) and lymph nodes (p=0.003) was found to be significant.

Among 65 patients whose clinical data were examined, MRI was present in 29 cases, CT in 21 cases, and both CT and MRI in 1 case in addition to ^18^F-FDG PET/CT.

### Comparative Evaluation of ^18^F-FDG PET/CT and MRI Findings

**Figure 2:**
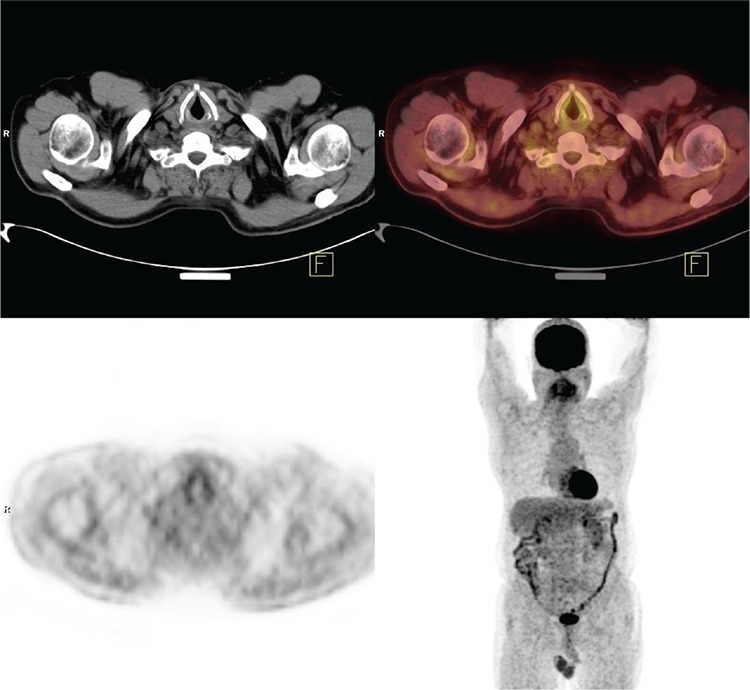
Patient with larynx squamous cell carcinoma. No pathologic findings with 18F-FDG positron emission tomography/computed tomography, left vocal cord histopathology report is compatible with carcinoma in situ

Within the group of patients with MRI (30 cases), 21 had pathological findings in primary lesion localization with MRI and PET/CT. Nineteen of these 21 patients were considered as true positive while the remaining 2 patients were accepted as false positive, according to their clinical follow-ups. Five of the 30 patients were negative on both MRI and PET/CT. According to histopathologic confirmation, two patients were accepted as false negative, one of them had spindle cell carcinoma on the floor of the mouth and the other one had larynx squamous cell carcinoma (Figure 2).

Lesions were observed in the clinical evaluation of two patients who had no pathological findings on MRI (false negative), but an increased metabolic activity was observed in the localization of the primary lesion on PET/CT (true positive). For the other two patients who had pathological findings on MRI and no hypermetabolic lesions on PET/CT, MRI was accepted as false positive according to their clinical follow-up.

In 5 of 14 cases, the pathological lymph node was not defined on MRI or PET/CT as confirmed by histopathologic biopsy (true negative in both examinations). In all the remaining 9 cases with histopathologically proven metastatic lymph nodes, hypermetabolic lymph nodes were defined on PET/CT (9 real positives), and in 8 of these patients pathological lymph nodes were demonstrated with MRI (8 true positives) while in 1 patient MRI could not detect the metastatic lymph node (1 false negative). PET/CT did not cause any change in the treatment protocol or in staging of this patient with a T3 tumor.

The sensitivity, specificity, positive predictive value (PPV) and negative predictive value (NPV) in evaluation of primary lesions and metastatic lymph node with MRI and ^18^F-FDG PET/CT are outlined in Table 2.

### Comparative Evaluation of ^18^F-FDG PET/CT and CT Findings

In the study group, 22 cases had a diagnostic neck CT. In a case with laryngeal carcinoma, despite asymmetrical thickening in the vocal cord, increased ^18^F-FDG uptake was not observed on PET/CT (false negative). The pathologic lesion was demonstrated in contrast-enhanced CT and then the presence of a tumor was verified histopathologically (true positive).

In another patient who had no local recurrence on PET/CT and no malignant lesion in histological examination, a pathologic finding was detected on contrast-enhanced CT (false positive).

Among the case group including 22 patients with neck CT and PET/CT, a metastatic lymph node was not observed on CT or PET/CT in 4 of 10 cases who had pathological confirmation (true negative). In 6 cases with metastatic lymph nodes on pathologic examination, PET/CT defined a pathological hypermetabolic lymph node (6 true positives), whereas CT did not define a metastatic lymph node in 3 of these 6 cases (3 true positives, 3 false negatives).

In the patients with a PET/CT and a contrast-enhanced CT, the results of sensitivity, specificity, PPV and NPV in primary lesion evaluation and metastatic lymph node determination are specified in Table 3.

### Contribution of ^18^F-FDG PET/CT in Staging and Re-staging of All Patients

PET/CT suggested the same disease stage with conventional imaging methods in 27 of 37 cases. However, in five cases who had suspicious findings with conventional methods, PET/CT indicated stage 2 (T2N0M0) disease in 2 patients, stage 4 (T4aNxM0, TxN2bM0) in 2 patients, and stage 3 (T3N1M0) in 1 patient, and it resulted in a change in staging (Table 4). In 25 of 28 cases who had conventional imaging, PET/CT imaging was obtained for re-staging purposes and it did not cause any change in ‘tumor, node, metastasis’ (TNM) classification.

## DISCUSSION

PET/CT plays a role in the treatment planning of HNCs by leading to a change in TNM classification, in especially locally advanced stage.

HNCs constitute 6% of all malignancies, and they are the 8th leading cause of cancer-related deaths (5). Correct staging is vital in treatment planning of HNCs.

In terms of T staging, PET/CT may be insufficient in determining tumor size, specifying the infiltration boundaries and invasion of the surrounding tissue. The presence of soft tissue, perineural and bone marrow invasion can be evaluated with MRI with a higher accuracy than PET/CT (6). However, recent studies have reported that in 1/3 of patients more accurate results can be obtained with PET/CT in terms of T staging (7). In our study, for evaluation of primary lesion; in the case group with MRI the sensitivity, specificity, PPV and NPV of PET/CT were found higher as compared to MRI; and in the case group with CT, the sensitivity and PPV of PET/CT were found to be higher than CT. However, ^18^F-FDG PET may not detect early-stage tumors with a small tumor volume, superficial tumors with a depth of less than 4 mm, as well as low stage tumors (such as carcinoma in situ) (8,9). In our study group, pathologic ^18^F-FDG uptake was not observed in tumors of two patients with larynx carcinoma (one with carcinoma in situ) along with another patient with spindle cell carcinoma of the oral cavity.

The presence of ipsilateral, contralateral or bilateral metastatic lymph nodes reduce 5-year survival rate of HNCs by about 50% (10). The presence of occult lymph node metastasis in cases who have been staged as N0 based on scanning techniques is reported to be approximately 25-30%. That is why several authors recommend routine neck dissection in each case (9). It is stated that in identifying metastatic lymph nodes PET/CT is superior to morphological scanning techniques with the advantage of functional information (11,12) in spite of the partial volume effect on the lymph nodes with millimetric sizes. In our study, in 4 of 24 patients (16.6%) who had PET/CT, CT, MRI imaging and histopathologically confirmed nodal metastasis, the hyper-metabolic lymph node was observed with PET/CT although there were no pathologic findings with conventional techniques. The false positive results obtained with PET/CT are usually related to reactive lymph nodes. Therefore, it is recommended that the lymph nodes with increased ^18^F-FDG involvement should be evaluated histopathologically (13). Schwartz et al. (14) reported the sensitivity of PET/CT and CT in detection of nodal metastasis as 96% and 78%, respectively. In our study, the sensitivity of PET/CT, MRI and CT in the evaluation of metastatic lymph nodes was calculated as 100%, 88.8% and 50% respectively.

In our study, the TNM stage according to PET/CT and conventional scanning techniques was the same in 27 of 37 patients. In five patients (13.5%), suspicious findings were identified with conventional techniques and the accurate result was obtained with PET/CT. In a prospective study by Lonneux et al. (15), it was stated that PET/CT may cause changes in treatment planning in 30% of patients with advanced stage head and neck squamous cell carcinoma, in 13% of patients with early stage disease, and in 13.7% of the entire group. PET/CT offers several advantages in planning of surgical treatment as compared to morphological scanning techniques by identifying the resection boundary of the primary lesion as well as the presence of lymph node metastasis (16). In addition, evaluation of distant metastasis with a single scanning technique and detection of a secondary malignancy is possible with PET/CT. The treatment options of surgery, systemic treatment and RT can be evaluated based on PET/CT data with much higher accuracy than conventional techniques and the risks related to an unnecessary radical surgical procedure or to inappropriate treatment can be avoided.

## CONCLUSION

The results of this study indicate that PET/CT provides more accurate staging of HNCs than conventional techniques, and it plays a significant role in treatment planning. PET/CT seems to produce similar results with conventional imaging methods in restaging of head and neck malignancies, due to the limited number of patients in the study group.

## Figures and Tables

**Table 1:**
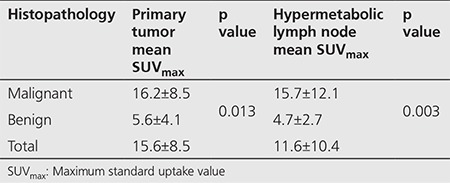
Correlation of maximum standard uptake value and histopathologic results in the primary lesion area and regional lymph node

**Table 2:**
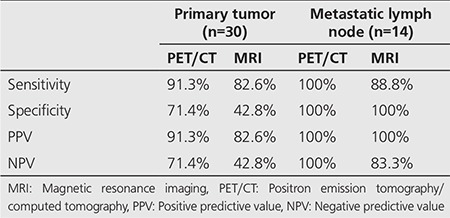
Diagnostic competence of positron emission tomography/computed tomography and magnetic resonance imaging in the evaluation of primary tumor and metastatic lymph node

**Table 3:**
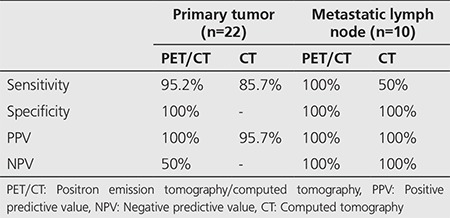
Diagnostic competence of positron emission tomography/computed tomography and contrast-enhanced computed tomography in the evaluation of primary lesion and metastatic lymph node

**Table 4:**
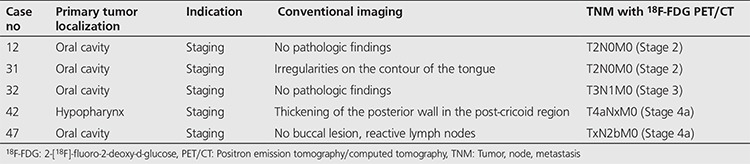
Tumor, node, metastasis staging with 18F-FDG positron emission tomography/computed tomography
